# Impact of Non-Tailored One-Way Automated Short Messaging Service (OASMS) on Glycemic Control in Type 2 Diabetes: A Retrospective Feasibility Study

**DOI:** 10.3390/ijerph17207590

**Published:** 2020-10-19

**Authors:** Ahmad Alamer, Charles Palm, Abdulaziz S. Almulhim, Charisse Te, Merri L. Pendergrass, Maryam T. Fazel

**Affiliations:** 1 Center for Health Outcomes and Pharmaco-Economic Research, University of Arizona College of Pharmacy, Tucson, AZ 85721, USA; 2 Department of Pharmacy Practice, College of Pharmacy, Prince Sattam Bin Abdulaziz University, Alkharj 16278, Saudi Arabia; 3 Banner—University Medicine Endocrinology and Diabetes Clinic, Tucson, AZ 85714, USA; charles.palm@bannerhealth.com (C.P.); cte@deptofmed.arizona.edu (C.T.); mpendergrass@deptofmed.arizona.edu (M.L.P.); maryamfazel@pharmacy.arizona.edu (M.T.F.); 4 Department of Pharmacy Practice, College of Clinical Pharmacy, King Faisal University, Al-Ahsa 31982, Saudi Arabia; asaalmulhim@kfu.edu.sa; 5 Department of Medicine, Division of Endocrinology, Diabetes and Metabolism, College of Medicine, Tucson, AZ 85724, USA; 6 Department of Pharmacy Practice & Science, College of Pharmacy, The University of Arizona, Tucson, AZ 85721, USA

**Keywords:** short messaging, SMS, non-tailored, one-way, automated, type 2 diabetes, uncontrolled diabetes, glycemic control

## Abstract

Short message service (SMS) is easily accessible and potentially an ideal platform for delivering patient-targeted messages. However, an effective SMS dosing strategy is not well established. Our purpose was to evaluate the impact of diabetes self-care promoting messages via non-tailored one-way automated SMS (OASMS) on glycemic control in type 2 diabetes (T2DM). The change in hemoglobin A1c (HbA1c) was compared between patients who received the service and those who did not. This retrospective quasi-experimental pre–post feasibility study was conducted at an academic medical center endocrinology clinic. English-speaking adults (≥18 years) with uncontrolled T2DM (HbA1c ≥ 8%) were included. A total of 69 patients (intervention *n* = 34; control *n* = 35) met the inclusion criteria. The mean (±SD) baseline HbA1c values were 10.2% (±1.9%) and 9.9% (±1.7%) in the intervention and control arms, respectively. Median follow-up was 3.3 months (IQR = 3–4.2). An ANCOVA model adjusted for baseline HbA1c and age showed an estimated HbA1c reduction difference of −0.97% (95% CI, −1.73 to −0.20%, *p* = 0.014), favoring the intervention arm. Inverse propensity score weighting confirmed the ANCOVA results. Our study suggests that adding diabetes self-care promoting messages via non-tailored OASMS to usual care improves glycemic control in poorly controlled T2DM. Larger and longer studies are needed to evaluate different features of the non-tailored OASMS strategy.

## 1. Introduction

On average, patients with diabetes spend less than 1% of their lifetime with their healthcare providers [1]. Self-care behaviors and problem-solving skills are important aspects of diabetes management. The American Diabetes Association (ADA) and American Association of Diabetes Educators (AADE) have introduced national standards highlighting the concept of Diabetes Self-Management Education and Support (DSMES) services. The goal of these services is to assist patients to gain the necessary diabetes self-care skills and knowledge, and to be able to implement behaviors to improve their disease management. Traditionally, these services were delivered via written materials or face to face [1,2]. Despite the proven benefits associated with these services, many high-risk individuals do not take advantage of those due to lack of perceived benefits and practical barriers such as transportation or work [3]. Mobile health (mHealth) is a healthcare platform that can increase convenience, access to health care and promote cost saving [4,5]. According to the Pew Research Center, the vast majority of Americans own cellphones with text messaging being the most popular service utilized across all age groups [6]. Given these trends, short message service (SMS) seems to be an ideal platform to deliver self-care education and support for diabetes patients. Thus, there is an increased interest in the literature to evaluate the efficacy of SMS on diabetes control. A study by Greenwood et al. showed that SMS was a feasible mean of delivering DSMES services and resulted in similar outcomes when compared to in-person visits [7].

Although the SMS intervention appears to be simple, it is important to consider the underlying complexity of such an intervention. The complexity arises from various aspects, including the behavioral change theory/technique used, materials, procedures, mode of delivery, frequency of the intervention over a specific period, timing, socioeconomic, cultural and health system factors [8]. The SMS mode of delivery can be automated versus non-automated, one-way versus two-way and tailored versus non-tailored. Investigators may mix these features to formulate an SMS dosing strategy. For instance, in tailored messaging, researchers may use patients’ clinical data to send personalized text messages that contain very specific instructions. In this scenario, adding a two-way messaging feature is desirable as it will provide a mechanism for patients to interact with their providers. This interaction is not feasible if an unidirectional (one-way) messaging from providers to their patients is used [9].

Globally, researchers have used different SMS dosing strategies to promote diabetes self-care behaviors and have shown mixed results in regard to glycemic control [10,11,12,13]. This has made the job of researchers evaluating these interventions difficult. A comprehensive systematic review identified seven randomized control trials examining the efficacy of automated text-messaging-based interventions on patients with poorly controlled T2DM. Three out of the seven trials reported a significant decrease in HbA1c. Due to variability in the methodology of these studies, authors were unable to conclude that the intervention had an impact on glycemic control [14]. A randomized non-blinded trial, not included in the previously mentioned systematic review, used a SMS intervention in Hispanic individuals with poorly controlled T2DM. The researchers used 2–3 messages per day with the frequency tapering over six months. The intervention demonstrated a significant reduction in HbA1c compared to a control group (*p* = 0.03) [14]. In this study, the investigators were allowed to call patients in case of hyperglycemia or hypoglycemia and this may have limited the ability to evaluate the SMS automated strategy.

To our knowledge, there are limited real-world studies that have evaluated a non-tailored one-way automated SMS (OASMS) strategy in patients with uncontrolled diabetes. We chose this strategy as its simplicity increases the potential for it being implemented in various settings. The aim of our study was to evaluate the impact of diabetes self-care promoting messages via non-tailored OASMS, when added to routine care, in patients with uncontrolled T2DM.

## 2. Materials and Methods

### 2.1. Study Setting and Design

This was a retrospective quasi-experimental feasibility study with a pre–post design. Due to the nature of the intervention, subjects and providers were not blinded during the study period. The setting was a single academic medical center endocrinology clinic in Tucson, AZ, USA. The study was approved by the University Institutional Review Board Human Subjects Protection Program.

### 2.2. Sample Selection

**Figure 1:**
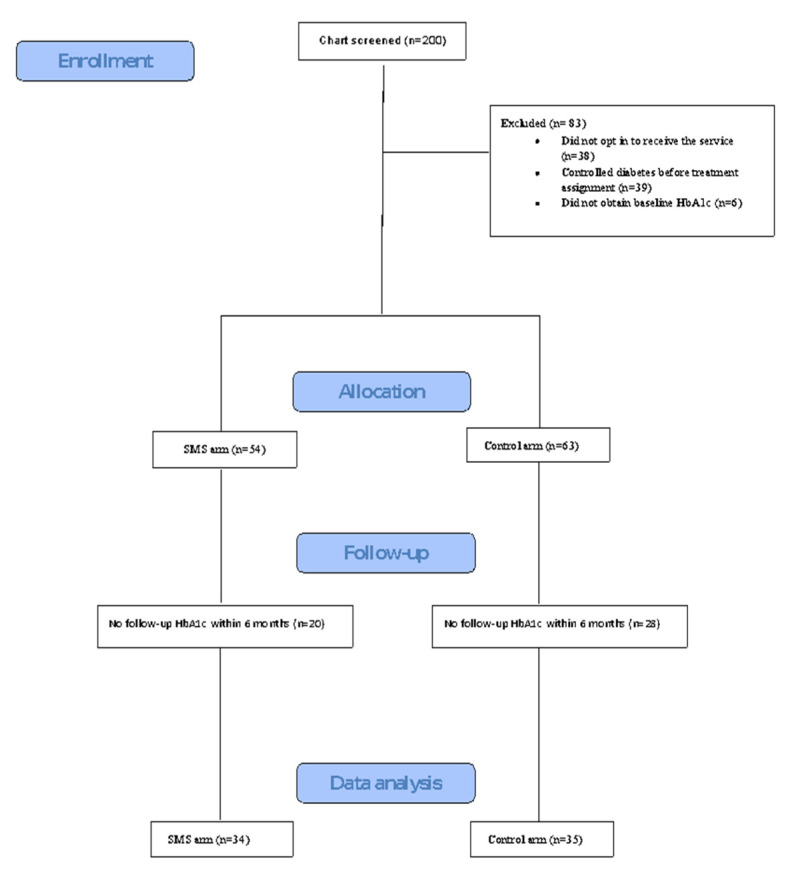
Flowchart of participant recruitment.

A convenience sample of patients seen in the clinic from 27 November 2017 to 3 May 2018 was screened for eligibility. Patients received the SMS intervention as part of an initiative program in the clinic intended to improve health care services and was offered by providers voluntarily. The control group was selected randomly from the list of patients seen in the pre-specified period. The following were the eligibility criteria: English-speaking adult patients (≥18 years) with uncontrolled T2DM, defined as hemoglobin A1c (HbA1c) ≥ 8%. We excluded patients who did not have a baseline (within 9 months pre-intervention) or follow-up (within 6 months post-intervention) HbA1c, or did not opt in to receive the service. See Figure 1 for the participant flowchart.

### 2.3. The SMS Intervention

Our dosing strategy was defined as the following: a non-tailored one-way automated SMS via text messaging. The SMS was set to be sent automatically at 10 a.m., hence “automated”. This strategy only allowed for unidirectional “one-way” messaging, in which there was no feedback mechanism in place. The intervention was not tailored/personalized to patients except for how many messages per week they preferred to receive (3 or 6 times a week). The content of these text messages was formulated by an interprofessional team that consisted of attending endocrinologists, Certified Diabetes Care and Education Specialists (a clinical pharmacist and a dietitian) and a health coach. The content of the SMS was designed to mirror the ADA/AADE recommended DSMES curriculum elements with a goal to promote behavioral changes and diabetes self-care in the patients. Multiple theories were elicited in this intervention including behavioral change technique theory [8]. For readability and understandability, test subjects received a sample of these messages and provided feedback. In total, there were 156 messages formulated with a maximum of 140 characters each. These were categorized broadly into: general diabetes knowledge, motivational messages, managing stress, nutritional facts, appointment reminders, medication adherence, monitoring reminders and complication management tips. See Table 1 for examples of the text messages. For reproducibility and transparency, we shared a full description of these text messages in the Supplementary Materials (File S1). Patients needed to opt in to receive the service and had the option to opt out at any time during the service period.

### 2.4. Data Collection and Variables

Demographic data, including age, sex, ethnicity, employment, insurance status and preferred language were collected. Pertinent baseline clinical data, including HbA1c, duration of diabetes, insulin use, weight (kg), body mass index (BMI) (kg/m^2^) and systolic/diastolic blood pressure were collected. Charlson Comorbidity Index (CCI) was calculated for each patient [15]. Data were manually extracted from the electronic medical record (Cerner^®^) and then entered in an unidentified manner into Research Electronic Data Capture (REDCap^®^), which is a web-based secure platform [16].

### 2.5. Outcomes

Primary outcome was the mean HbA1c reduction in both arms within 6 months after the start of the intervention. Secondary outcomes were the median number of clinic visits and percentage of patients who opted out of the service.

### 2.6. Statistical Analysis

Data were analyzed using RStudio (2019) software (R Foundation for Statistical Computing, Version 3.6.0, Vienna, Austria). Descriptive and inferential statistics were used to analyze the data. Continuous and categorical variables were described as means ± standard deviation (SD), median interquartile range (IQR) and percentages, respectively. Chi-square test was used for categorical data, Student *t*-test for means and Mann-Whitney U for medians. For the HbA1c outcome, analysis of covariance (ANCOVA) was used to adjust for baseline HbA1c and age variables [17]. Normality and linearity assumptions were tested for all models using diagnostic plots (normal Q–Q plot and residuals plots) and homogeneity of residuals (constant variance across arms) was tested using Levene’s test. All *p*-values less than 0.05 were considered statistically significant. For visual representation, ggplot2 package was used in the RStudio interface [18].

### 2.7. Propensity Scores and Inverse Propensity Score Weighting (IPSW) 

Due to the small sample size, propensity scores matching procedure was not feasible. Alternatively, to circumvent the non-randomized design of the study and account for additional confounders on the outcome of interest, we estimated propensity scores for treatment assignment. The following covariates were considered in the propensity score calculation (age, baseline HbA1c, ethnicity, employment status, type of insurance, diabetes regimen, diabetes duration, BMI and CCI). Using propensity scores, we calculated the inverse propensity score weights (IPSW) using the “ipw” package in R [19]. Using these weights, we then fitted a marginal structural model to estimate the causal effect of the intervention on HbA1c reduction. The IPSW analysis is meant to confirm the results of ANCOVA model by adjusting for additional confounders. It has been shown using propensities as weights produces more reliable results in small sample sizes as compared to matching procedures [20].

### 2.8. Response (HbA1c Reduction) Predictors

As an exploratory secondary analysis, we performed linear regression models (both univariable and multivariable) to identify baseline variables associated with HbA1c reductions. For the multivariable linear regression, we implemented a “backward elimination” stepwise methodology to select the best predictors for the HbA1c reduction response.

## 3. Results

Two hundred patient charts were screened. A total of 69 patients met the inclusion criteria for our analysis. Of those, 34 patients received SMS in addition to usual care (intervention arm) and 35 received usual care (control arm). Figure 1 specifies reasons for exclusion. The two most common reasons for exclusion were missing baseline or follow-up HbA1c.

**Figure 2:**
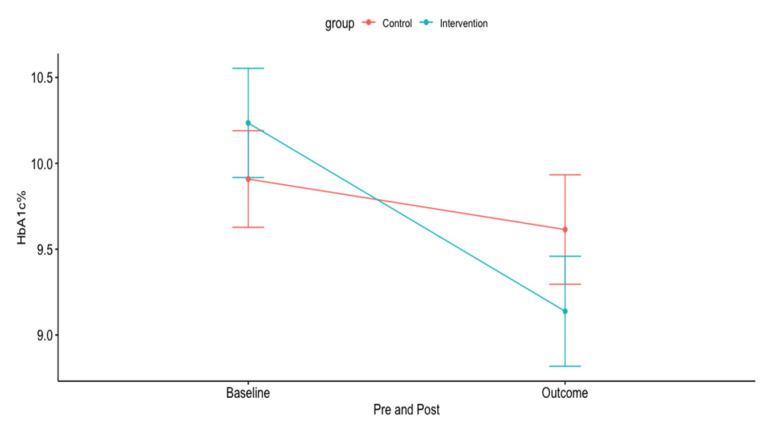
Pre-post hemoglobin A1c (HbA1c) reduction in the control and intervention arms.

Baseline characteristics are shown in Table 2. Overall, baseline characteristics did not differ significantly between the arms, except for ethnicity and the mean age. The mean baseline HbA1c was about 10% in both groups with a median post-intervention follow-up of 3.3 months (IQR = 3 to 4.2 months). The mean (±SD) HbA1c outcome in the intervention arm was 9.14% (±1.87%) versus 9.61% (±1.89%) in the control arm (Table 3). Patients in the intervention arm had a greater crude HbA1c reduction from baseline with a mean reduction of −1.1% (95% confidence interval [CI], −1.8 to 0.4%) compared to −0.3% (95% CI, −0.7 to 0.1%) in the control arm (shown in Figure 2, Table 3). After conducting ANCOVA adjusted for age and baseline HbA1c as covariates, the results showed a significant effect for arms with F (1, 65) = 6.36, *p* = 0.014. The estimated difference in HbA1c reduction was −0.97% (95% CI, −1.73 to −0.20%, *p* = 0.014) between the intervention and control arms, favoring the intervention arm (Table 3). The estimated marginal causal effect of the intervention on HbA1c using IPSW was −0.98% (95% CI, −1.88 to − 0.08%, *p* = 0.036) confirming the results from the ANCOVA model. The results of the exploratory multivariable linear regression are shown in Table 4 and those of the univariable linear regression analysis in the Supplementary Materials Table S1. The most important predictors for the HbA1c were age, baseline HbA1c and the OASMS intervention. The secondary outcomes are presented in Table 5. There was a statistically significant increase in the number of clinic visits for the control arm compared to the OASMS arm. Two patients opted out of the service in the intervention arm.

## 4. Discussion

Our study demonstrated a greater reduction in mean HbA1c from baseline in patients with uncontrolled T2DM who received non-tailored OASMS intervention in addition to usual care when compared to those receiving usual care alone. We recognize that randomized control trials (RCTs) remain the gold standard to evaluate interventions. However, our findings add real-world evidence of the effectiveness of this intervention as an inexpensive and convenient approach to delivering diabetes self-care promoting messages in patients with uncontrolled T2DM. The dosing strategy we adopted in this study is one of the simplest strategies reported in the literature [9,18]. The messages were automated, non-tailored and unidirectional and therefore were minimally labor intensive.

A recent meta-analysis by Zhuang et al. evaluated 10 clinical trials that examined SMS interventions as a tool to improve glycemic control in patients with T2DM. Similar to our study, most of the trials utilized one-way automated messages to deliver educational material and/or medication adherence reminders. Mean HbA1c was reduced by −0.49% (95% CI, −0.75 to 0.22%) in the intervention arm. It is worth noting that there was heterogeneity in the results (I^2^ = 64.90%, *p* = 0.002) that can be explained by differences in features of the SMS dosing strategy such as terms of frequency, content of the messages, duration and clinical setting. [9]. Another meta-analysis by Sahin et al. analyzed 11 trials to examine this approach. They showed that the impact on glycemic control was not different when the tailored and non-tailored features were compared; however, a sub-group analysis revealed that non-automated messages produced larger HbA1c effects than automated messages (*p* < 0.001). Interestingly, one-way messaging (*p* < 0.022) and infrequent messaging (*p* < 0.001) were more effective than two-way messaging [21]. A key difference between the two mentioned meta-analyses and the one by Dobson et al. is that the latter examined the efficacy of SMS intervention on patients with poorly controlled T2DM [14]. Upon close examination, the investigators found mixed evidence with regards to the intervention in this population. Only two out of seven RCTs in the systematic review specifically targeted patients with poorly controlled T2DM, defined as HbA1c ≥ 8%, which is similar to the inclusion criteria in our study. Only one of the two RCTs (Arora et al.) had a fairly similar intervention to ours; however, it had a higher frequency SMS intervention (2 times a day) but did not reach statistical significance in their primary outcome of HbA1c reduction (–0.45%, 95% CI, –0.27 to 1.17%; *p* = 0.24) with greater reduction seen in Hispanic patients [22]. A later study by Fortmann et al., “The Dulce Digital study”, was conducted in Hispanic patients with a similar sample size to the study by Arora et al. (*n* = 64 in each arm) found a statistically significant reduction in HbA1c compared to usual care (*p* = 0.03) [23]. Notably, the “Dulce Digital study” allowed investigators to call patients in the case of hyperglycemia or hypoglycemia, which limited the reliability of their results on evaluating the automation and unidirectional features of their SMS intervention.

While our study used a non-tailored OASMS dosing strategy, several researchers have examined tailored messaging features to deliver SMS [9]. The content of messages may differ and can include specific/tailored instructions on medications (e.g., dose adjustments), or a feedback mechanism (two-way messaging) where providers can interact with their patients. Given the heterogeneity in the SMS dosing strategies described in the literature, it would be very difficult to delineate an ideal SMS dosing formula that works in every setting. Moreover, it appears that there is limited evidence in the literature supporting the use of non-tailored OASMS in poorly controlled T2DM [14]. In addition, the majority of the published studies that reported the efficacy of SMS did not share their full SMS formula to allow for independent investigations [14]. On the other hand, we believe in open source science and methodology; thus, we shared our full SMS content formula in the Supplementary Materials to provide means for reproducibility by future investigators. The non-tailored OASMS dosing strategy can benefit from large studies that evaluate different features (such as SMS frequency) and their impact on glycemic control. Due to the sample size we were not able to run additional analyses on adequate OASMS frequency in the current study. With regards to the secondary outcomes, we noted a statistically significant increase in the number of clinic visits in the control arm. One explanation could be that patients in the control arm required more clinic visits for medication adjustments to meet their HbA1c goals. Notably, more visits in the control arm would mean more interventions being made, potentially skewing the results in favor of the control. However, our results showed that the intervention arm had a greater reduction in HbA1c. Another explanation may be that the intervention indeed had an impact on patients’ diabetes self-care behaviors leading to less need for clinic visits. Future studies will need to evaluate this.

There are some limitations in our study. The retrospective, non-randomized and non-blinded design of the study can affect our conclusion about the true effect of the intervention. Non-randomization is a known issue with mHealth interventions in general [14]. To deal with this design issue and minimize bias, we calculated propensity scores for treatment assignment and used the IPSW to estimate the marginal causal effect of the treatment. The result of this analysis confirmed the result of the ANCOVA model. Moreover, the exploratory multivariable linear regression revealed the most important predictors were age and HbA1c at baseline, both of which were considered in the ANCOVA model. Since this was a feasibility study, we did not initially perform sample calculations; however, based on the effect size obtained, post-hoc calculation suggests that the study achieved the power needed to detect difference between two the arms (power > 90%). Reasons for loss to follow-up are presented and were mostly due to missing baseline or follow-up HbA1c. Furthermore, our subjects were limited to English-speaking individuals and we may have missed high-risk minority patients who do not have access to mobile services. Given that our median follow-up for HbA1c data was about 3 months, it is unknown if HbA1c reductions would be sustained beyond 6 to 12 months. Lastly, we were not able to assess adherence and diabetes self-care behavioral changes during the study period.

## 5. Conclusions

This study demonstrated real-world effectiveness of delivering diabetes self-care promoting text messages via non-tailored OASMS to patients with uncontrolled T2DM. We believe that there was a promising and clinically relevant reduction in HbA1c from baseline in the intervention group compared to the control. However, this intervention will need to be evaluated in larger clinical trials to examine the impact of different formulas (dosing) of OASMS on glycemic control. Meanwhile, clinics may consider using the service for their patients. It is important to note that the use of this service, due to its non-tailored and one-way characteristics, should be considered supplemental to ADA/AADE standardized DSMES services.

## Figures and Tables

**Table 1:** Examples of the non-tailored automated one-way SMS content. A full description can be accessed in the Supplementary Materials (File S1).

Diabetes Knowledge
Many people need insulin when their blood sugar is high but can stop taking it later–especially if they have lost weight. Diabetes Fact: Glucose, the sugar in your blood, is your main energy source–sort of like the gas in your tank.
**Motivation**
Remember, taking care of your diabetes has ups & downs. Don’t focus on one thing. Look at trends over time. Most people sometimes forget to take meds. But taking meds is the MOST important step you can take to stay healthy!
**Managing Stress Tips**
Feeling stressed? Sit quietly, close your eyes, breathe deeply, count as you exhale. Do this 10 times & feel the difference! Relax! Take a mini-vacation by sitting quietly, closing your eyes, and just breathing for a minute.
**Nutritional Facts**
You can still eat out! Order smaller items from a fast food menu. Even small changes help control diabetes! Shop smart! On a food package nutrition label, look at how many “Servings Per Container” there are.
**Monitoring Reminders**
Be a scientist! Check your blood sugar before & after exercise to see how the exercise affects the results. If you check your blood sugar at home, keep a log & show it to your doctor. She will use the info to adjust your medication doses.
**Taking Medications**
Don’t miss out on better health. Take your prescribed meds today! Don’t skip your meds! If you have trouble affording prescriptions, there may be cheaper options. Talk to your doctor.
**Managing Complications**
Reminder: Getting your eyes checked every 1 to 2 years can reduce the risk of blindness! Check your feet every day. Let your health care provider know if you develop any sores or ingrown toenails.

**Table 2:** Baseline characteristics.

Characteristic	OASMS Arm *n* = 34	Control Arm *n* = 35	*p*-Value
Age, (years), mean (±SD)	53.3 (11.8)	61 (13)	0.012
Male, *n* (%)	14 (41.2)	19 (54.3)	0.396
Ethnicity, *n* (%)			0.007
White	18 (52.9)	27 (77.1)	
African American	0 (0)	3 (8.6)	
Native American	1 (2.9)	0 (0)	
Hispanic	13 (38.2)	5 (14.3)	
Other/unknown	2 (5.9)	0 (0)	
Employment, *n* (%)			0.581
Employed	5 (14.7)	2 (5.7)	
Non-employed	10 (29.4)	12 (34.3)	
Disabled	1 (2.9)	0 (0)	
Retired	5 (14.7)	8 (22.9)	
Unknown	13 (38.2)	13 (37.1)	
Preferred language, *n* (%)			
English	34 (100)	33 (97.1)	1.000
Insurance, *n* (%)			0.256
Medicare	7 (20.6)	8 (22.9)	
Medicaid	11 (32.4)	17 (48.6)	
Commercial	16 (47.1)	10 (28.6)	
Duration of diabetes, years, mean (±SD)	14.2 (10.8)	16.3 (12)	0.445
Diabetes regimen, *n* (%)			0.479
Insulin only	6 (17.6)	7 (20)	
Non-insulin therapy	9 (26.5)	6 (17.1)	
Insulin combined with non-insulin therapy	18 (52.9)	17 (48.6)	
Insulin pump	1 (2.9)	4 (11.4)	
No medications	0 (0)	5 (2.9)	
Charlson comorbidity score, mean (±SD)	3 (1.7)	3.8 (2.1)	0.633
HbA1c %, mean (±SD)	10.2 (1.9)	9.9 (1.7)	0.673
Systolic blood pressure, mm Hg, mean (±SD)	136.8 (13)	134.2 (15.5)	0.454
Diastolic blood pressure, mm Hg, mean (±SD)	81 (12.4)	75.6 (11.6)	0.059
Weight, Kg, mean (±SD)	102.4 (26.7)	99.1 (32.9)	0.673
Body mass index (BMI), kg/m^2^, mean (±SD)	38.6 (15.2)	34.2 (9.8)	0.162

OASMS: One-way automated short messaging service. HbA1c: hemoglobin A1c.

**Table 3:** Crude and adjusted HbA1c outcomes.

Variable	OASMS Arm *n* = 34	Control Arm *n* = 35
HbA1c % outcome, crude mean (±SD)	9.14 (1.87)	9.61 (1.89)
HbA1c %, outcome adjusted means (95% CI) ^†^	8.89 (8.36 to 9.42)	9.85 (9.33 to 10.37)
HbA1c %, crude mean reduction from baseline (95% CI)	−1.1 (−1.8 to –0.4)	−0.3 (0.7 to 0.1)
HbA1c %, adjusted mean reduction from baseline (95% CI) ^†^	−1.17 (−1.71 to −0.64)	−0.21 (−0.73 to 0.31)

HbA1c: hemoglobin A1c; OASMS: one-way automated short messaging service; ^†^ Result for the ANCOVA analysis (adjusted for baseline and age) for OASMS versus control with estimated HbA1c reduction of −0.97% (95% CI, −1.73 to −0.20%, *p* = 0.014); effect size (r) = 0.39.

**Table 4:** Multivariable linear regression for the most significant predictors of HbA1c reduction.

Characteristic	Estimate (95% CI)	*p*-Value
Intercept	7.411 (4.058–10.763)	<0.001
Age (years)	−0.042 (−0.073 to −0.011)	<0.001
HbA1c at baseline	−0.517 (−0.738 to −0.295)	<0.001
OASMS Intervention	−0.965 (−1.729 to −0.200)	0.014

OASMS: one-way automated short messaging service. HbA1c: hemoglobin A1c. Adjusted R squared 0.271; *p*-values are derived from t-statistic; F-statistic: 9.449 and *p*-value for the model <0.001.

**Table 5:** Secondary outcomes.

Variable	OASMS Arm *n* = 34	Control Arm *n* = 35	*p*-Value
Number of clinic visits, median (IQR)	3 (1–3)	3 (2.5–4)	0.011
Text received, median (IQR)	57.50 (36–78)	-	NA
Opted out of service, N (%)	2 (5.9)	-	NA

OASMS: one-way automated short messaging service. IQR = interquartile range. NA = not applicable.

## Supplementary Materials

The following are available online at https://www.mdpi.com/1660-4601/17/20/7590/s1, File S1: SMS full description and supplementary analyses.
