# Supplementary material for: Impact of Non-Tailored One-Way Automated Short Messaging Service (OASMS) on Glycemic Control in Type 2 Diabetes: A Retrospective Feasibility Study
Source: Int J Environ Res Public Health. 2020 Oct 19;17(20):7590. doi: 10.3390/ijerph17207590 (PMC7593936; doi:10.3390/ijerph17207590)
Supplement: Supplementary file 1 [file ijerph-17-07590-s001.zip › Supplemental material 1.docx]

| **Table S1. Univariable linear regression for predictors explaining HbA1c reduction** | | | |
| --- | --- | --- | --- |
| **Characteristic** | **Estimate(95%CI)** | **P-value** | **Adjusted R squared** |
| Age (years) | -0.008(-0.041 to 0.024) | 0.604 | -0.011 |
| Female | 0.321(-0.535 to 1.17) | 0.457 | -0.006 |
| Ethnicity |  |  | -0.014 |
| White | Reference | - |  |
| Non-white | 0.480(-0.463 to 1.424) | 0.313 |  |
| Unknown | 0.037(-2.092 to 2.167) | 0.971 |  |
| Employment, N (%) |  |  | -0.010 |
| Employed | Reference | - |  |
| Non-employed | -0.418(-1.838 to 1.00) | 0.559 |  |
| Insurance |  |  | 0.007 |
| Medicare | Reference | - |  |
| Medicaid | 0.856(-0.274 to 1.986) | 0.135 |  |
| Commercial | 0.573(0.573 to 1.489) | 0.551 |  |
| Duration of diabetes (years) | 0.012(-0.025 to 0.051) | 0.519 | -0.009 |
| Diabetes regimen, N (%) |  |  | -0.016 |
| Insulin only | Reference |  |  |
| Insulin combined with non-insulin therapy | -0.485(-1.522 to 0.551) | 0.353 |  |
| Non-insulin therapy | -0.187(-1.415 to 1.040) | 0.762 |  |
| Charlson comorbidity score | -0.098(-0.318 to 0.120) | 0.371 | -0.003 |
| HbA1c % at baseline | -0.439(-0.661 to -0.216) | <0.001 | 0.176 |
| Body mass index (BMI) (kg/m2) | 0.016(-0.013 to 0.054) | 0.225 | 0.007 |
| Group |  |  | 0.038 |
| Control | Reference |  |  |
| SMS arm | -0.802(1.640 to 0.034) | 0.060 |  |
| SMS: One-way Automated Short Messaging Service. HbA1c: Hemoglobin A1c. | | |  |
